# Mixed Methods in Tactical Analysis Through Polar Coordinates and Function Estimation: The Transition Play in ACB Basketball

**DOI:** 10.3389/fspor.2021.739308

**Published:** 2021-09-23

**Authors:** José Luis Pastrana-Brincones, Belén Troyano-Gallegos, Juan Pablo Morillo-Baro, Raimundo López de Vinuesa-Piote, Juan Antonio Vázquez-Diz, Rafael E. Reigal-Garrido, Antonio Hernández-Mendo, Verónica Morales-Sánchez

**Affiliations:** ^1^Department of Languages and Computer Science, University of Málaga, Málaga, Spain; ^2^Department of Social Psychology, Social Work, Anthropology and East Asian Studies, University of Málaga, Málaga, Spain

**Keywords:** mixed method, systematic observation, polar coordinates, basketball, prediction

## Abstract

Nowadays, getting advantageous offensive situations in high-level basketball is being increasingly harder, so taking advantage of any situation in the game since the team has the ball is essential to be competitive. Therefore, the goal to achieve in this study is to evaluate using a mixed method strategy the behaviors happening in the application of the technical–tactical means performed in the transition play of professional basketball in Spain. An *ad hoc* observation tool made of 11 criteria and 83 exhaustive and mutually exclusive categories (E/ME) has been designed and validated by means of data quality and generalizability analyses. Indexes obtained show high reliability and validity allowing the proposed actions to be recorded (correlation coefficients are above 0.95 and generalizability coefficients are above 0.90 in all cases). A total number of 128 situations corresponding to eight games of Unicaja de Málaga in the Endesa League in the 18/19 season were observed with the Hoisan software. The analysis of the relationships among behaviors was performed using the polar coordinates technique where the one-on-one initiation, outside the zone, has been used as focal behavior. The estimation of the functions representing the vectors has also been performed to model the best fit that estimates, starting from a focal category, the relationship among this focal behavior and the rest of the mating behaviors for possible future observations. The results show significant relationships between the selected focal behavior and the mating behaviors, showing indications of behaviors allowing tactical interpretation of the game and the definition of intervention programs to improve the performance of the team.

## Introduction

Basketball is a team sport, where interactions occur with both teammates and opponents. Therefore, although it is a sport that has been studied from many different disciplines such as biomechanics (Morales-Toapanta et al., 2018), psychology (Rodríguez-López and Sáez-Rodríguez, 2009), or physiology (Calleja et al., 2008), it must also be analyzed from a perspective where motor interaction is taken into account (Hernández-Mendo et al., 2000) because the player and his/her technical–tactical reading are the protagonists.

Nowadays, basketball is more physical, technical, tactical, and studied by coaches than before, and it is increasingly harder to get offensive advantages that allow to score points, so we try to take advantage of any situations in the attack phase from the first moment when the team has the ball. Most authors (De Torres and Arjonilla, 1999; Carballo and Dopico, 2005; Bazanov et al., 2006) differentiate three ways of attacking: counterattack, transition, and positional attack.

Generally called as transition play, the second wave of counterattack or as defined by the National School of Coaches (2010) play on, it involves those intermediate movements that a team performs after a direct counterattack is not completed, keeping the control of the ball. It arranges the players in the appropriate position on the field to initiate some movements, previously established by the coach, and without stopping the ball, with the aim of surprising and creating an offensive advantage. Individual and/or collective technical-tactical effectiveness makes that going along with the defense, carrying out the defensive balance, becomes as transcendental in high competition sport (Lozano, 2014).

Transitions are one of the least studied tactical aspects in basketball as they only represent approximately from 7 to 12% of all possessions in a game (Cruz and Tavares, 1998; Fernandes and Tavares, 2004). These play situations require the athlete to take decisions quickly, processing intuitively, automatically, and unconsciously the information coming from the environment (Kahneman, 2011). As they are unpredictable situations, it makes them difficult to defend, so improving this facet of the game using incidental strategies by manipulating contextual variables could make teams be more competitive (Camacho-Lazarraga, 2016).

One of the most recurrent offensive tactical means is the direct block (Nunes et al., 2016; Romarís-Durán, 2016), defined as the collective action of the two-on-two (2 × 2) play, where the attacker without the ball performs a block (hinders an opponent by placing himself/herself in his path) helping to his/her mate who is having the ball (Cárdenas, 2010; Muñoz-Arroyave et al., 2015; Nunes et al., 2016). It is so important that it is the most used action to end attack situations in ACB League (Romarís Durán et al., 2013). On the other hand, the one-on-one (1 × 1) is present in 58% of attacks finished outside the zone and 23% of attacks finished inside the zone (Lehto et al., 2010). Therefore, its study added to other tactical means is extremely interesting for coaches who aim to optimize their team play (Nunes et al., 2016).

Nowadays, using Observational Methodology (OM) in high-performance sport links in an optimal way the relationship between science and its practical application because it is applied on its natural context, being focused on the spontaneous and habitual behavior of the observed participants (Anguera, 1990; Anguera and Hernández-Mendo, 2013; Sánchez-Algarra and Anguera, 2013), such as competition. In addition to the possibility of developing an *ad hoc* observation instrument (Sarmento et al., 2010) adapted to the reality of a given context, it allows a detailed analysis of the behaviors involved in the tactical development of the game.

OM has been successfully applied in basketball (Hernández-Mendo et al., 2000; Romarís Durán et al., 2013; Muñoz-Arroyave et al., 2015), highlighting the impact of using polar coordinate analysis as a specific technique for studying the relationships established among the behaviors happened (Nunes et al., 2016). It is a technique that has also shown useful for other collective sports studies as taken for soccer (Echeazarra-Escudero et al., 2015; Castañer et al., 2016, 2017; Maneiro and Amatria, 2018; Maneiro et al., 2019), handball (Sousa et al., 2015; Morillo-Baro et al., 2017; Jiménez-Salas et al., 2020a,b; Quiñones et al., 2020), or beach handball (Morillo-Baro et al., 2015; Vázquez-Diz et al., 2019a,b).

The polar coordinate technique uses a sequential prospective and retrospective lag analysis of recorded behaviors (Sackett, 1980; Anguera, 1997; Anguera et al., 1997). It allows a great reduction in the amount of the analyzed data as well as in the graphical representation of the relationships established between focal and conditioned categories in a system of vectors (Hernández-Mendo and Anguera, 1999). The contrast statistic of this analysis is the Zsum (Zsum = Σ*z*/√*n*, where *n* is the number of lags) (Cochran, 1954). The distribution of this Zsum parameter has a x = 0 and an S*x* = 1. The relationships among behaviors and their vector representation are obtained from those values. A value is considered statistically significant when the module of the vector is equal to or greater than 1.96. This value is obtained by the square root of the addition of the square of the *Z*sum of *X* (prospective) and the square of the *Z*sum of *Y* (retrospective):


(1) $$\begin{array}{r} Module=\sqrt{ZsumP^{2}+ZsumR^{2}} \end{array}$$

The angle of the vector (ϕ = *Arc sine of Y/Radius*) will determine the excitatory or inhibitory nature of the relationship (Castellano and Hernández-Mendo, 2003).

Considering that background, the aim of this study is to evaluate using polar coordinates analysis the behaviors happening in the application of the individual technical–tactical means one-on-one in the transition play of professional basketball teams in Spain. And subsequently, make predictions using different statistical modeling techniques in order to estimate the best model explaining the relationship between focal behaviors and mating behaviors.

## Materials and Methods

The design of this study was placed in the fourth quadrant (Anguera et al., 2011); defined as follow-up, nomothetic, and multidimensional.

### Participants

Unicaja De Málaga team from the Spanish ACB Endesa League has been chosen for this study, a total of eight matches have been observed where a total of 128 analysis situations have been obtained in the 2018/2019 season. The number of games has been estimated through a generalizability analysis.

As UNICAJA ends the 18/19 season in Play Off place, it has been decided to make the analysis using regular season matches against opponents that have ended that season in Play Off qualifying places, so they would have a similar level (Table 1).

**Table 1:** Dates, results, and games played by Unicaja Baloncesto.

**Date**	**Party**	**Result**
28/09/18	**Unicaja**—Valencia	86–73
16/12/18	**Unicaja**—Barcelona	78–73
23/12/18	**Unicaja**—Baskonia	64–81
27/1/19	Barcelona—**Unicaja**	94–83
7/4/19	**Unicaja**—Juventut	88–63
24/4/19	Valencia—**Unicaja**	96–57
3/2/19	**Unicaja**—Real Madrid	103–102
9/2/19	**Unicaja**—Manresa	99–97

The observations have been taken from official competitions and belonging to publicly available videos and so, in accordance with the Belmont Report (1) and the competition rules (2), as the videos analyzed for this study are in a public domain, it is not necessary to obtain an informed consent from the participants. The Belmont Report describes the basic ethical principles and guidelines regarding ethical issues in human research. According to those guidelines, images of public behaviors can be used for researching without the informed consent of the athletes. In addition, neither a review by a research ethics committee nor a written informed consent is required in this study because of the following reasons: (a) it involves observation of people in public places (stadium); (b) the individuals or groups being observed have no reasonable expectation of privacy; (c) it does not involve any staged intervention by the researcher or direct interaction with individuals (https://student.societyforscience.org/human-participants).

### Instruments

Hoisan software (Hernández-Mendo et al., 2012a, 2014) was used in this study in order to code and record the behaviors, perform the data quality analysis and the polar coordinates analysis. The optimization of the polar coordinates analysis graphical representation has been performed with the R program (Rodríguez-Medina et al., 2019). Generalizability analysis has been done by the Software for Generalizability Theory analysis (SAGT) program (Hernández-Mendo et al., 2012b, 2016).

Previously, an *ad hoc* observational instrument has been developed to code the behaviors occurring in offensive transition situations. Table 2 shows the criteria and categories of the multidimensional design created.

**Table 2:** Observation instrument for the tactical evaluation of transition play.

**Criteria**	**Categories**	**Description**
Fourth	1C	First
	2C	Second
	3C	Third
	4C	Fourth
	5C	Extension
Zone	TLD	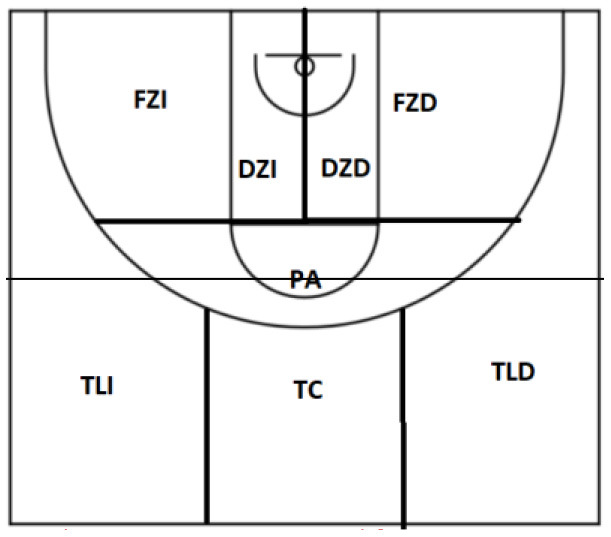
	TC	
	TLI	
	PC	
	FZI	
	FZD	
	DZI	
	DZD	
Location	L	Local
	V	Visitor
	N	Neutral
Marker	GM7	Winning + 7
	GU7	Winning 7 or –
	PM7	Losing + 7
	PU7	Losing 7 or –
	E	Tie
Defensive Balance	AJ	Adjusted
	DJ	Misaligned
1st Medium	PR	Pick and Roll
Tactical	PP	Pick and Pop
Collective	UxE	1x1 Outdoor
	UxI	1x1 Interior
	BIE	Indirect External Blocking
	BII	Indirect Blocking to Interior
	PAT	Back door
	FBD	False side BD.
	OMT	Another Media Tac. Col.
	PS	Pass
	MM	Hand in hand
Player Creates	BV	Point guard
Advantage	EV	Shooting guard
	AV	Small forward
	APV	Power forward
	PV	Center
Collaborators	BC	Point guard
	EC	Shooting guard
	AC	Small forward
	APC	Power forward
	PC	Center
	NC	Nobody collaborates
	EIC	Exterior blocks interior
	IEC	Indoor blocks outdoor
	EEC	Exterior blocks exterior
	IIC	Interior blocks interior
Defensive	FPP	Big Flash, Small chase
Action	PPA	G Push, P Back.
	APP	G Back, P Pursue.
	APD	G at the back, P at the back.
	APML	G back, P sends side
	CW	Defensive change
	UXU	One by one
	ETD	Defensive tactical error
	NXT	Def. Next
	OAD	Other Acc. defensive
Continuation	JLV	Cont. free play advantage
of the Play	JLN	Cont. J. Free No advantage
	JT	Cont. tactical play
	PP	Loss pass
	PB	Lost boat
	PR	Loss reception
	TS	Solo shooting
	TO	Opposition shooting
	PV	Advantageous pass
	PNV	Non-Advantageous pass
	FA	Foul on attack
	FD	Foul in defense
	OC	Other continuations
Final	AT2	Hit Shot 2
	ET2	Error Shot 2
	AT3	T3 Hit
	ET3	Error Shot 3
	MP	Maintains possession
	CP	Change of possession
	FAC	Missing attack cont.
	FDC	Lack of defense cont.
	OF	Other finals

The observational instrument passed the data quality tests required by observational methodology. Table 3 shows Kendall's, Pearson's, and Spearman's Tau *b* correlation coefficients results, reaching minimum indexes of 0.952 and Cohen's Kappa index shows a minimum value of 0.962.

**Table 3:** Values of Kendall's, Pearson's, and Spearman's Tau b correlation coefficients.

**Whole session coefficient**	**Intra (Obs. 1 vs. Obs. 1 bis)**	**Concordance Inter (Obs. 1 vs. Obs. 2)**
**Correlation coefficients and concordance index**		
Kendall's Tau b	0.986	0.977
Pearson	0.991	0.993
Spearman	0.994	0.952
Cohen's Kappa	0.981	0.962

The generalizability theory (Cronbach et al., 1972; Cardinet et al., 1976, 1981) allows to control the adequate quality of the data coming from the Observational Methodology, setting an accurate estimation of the different sample sizes (Blanco-Villaseñor et al., 2014).

In the interobserver reliability estimation, a two-facet crossover design, categories and observers (C/O), has been used. The results obtained by the SAGT program show that the variability is almost entirely associated with the (categories) facet (98.57%), being 0 in the (observers) facet and 1.42% in the (categories/observers) interaction facets. The generalizability coefficients estimated in this design structure are 0.99, for both the relative G coefficient (reliability) and the absolute G (generalizability) coefficient, which is considered as excellent results. A similar design has been performed for the intraobserver reliability calculation, and the results indicate that the variability is associated with the (categories) facet in 99.15%, being 0 in the (observers) facet and 0.84% in the (categories)/(observers) interaction facets. The results obtained are optimal because the two coefficients of generalizability, relative G and absolute G, are at 0.99.

A two-facet observer–category (O/C) crossover design has been used to calculate the fit and homogeneity of the categories in the sense of differentiators. The generalization coefficients in this measurement design are 0 (0.00). The 98.57% of variance is applied to the (categories) facet, being 1.42% in the (observers/categories) interaction and 0 in the (observers) facet. As soon as the generalization coefficients approach to 0, the fit and homogeneity of the categories, in the sense of differentiators, can be considered as optimal (Blanco-Villaseñor et al., 2014).

Categories and matches (C/P) have been chosen for estimating the minimum number of sessions needed to generalize accurately the two-facet design. The evaluation of the variance components has been performed in an infinite randomized manner for categories and matches. The measurement study result is shown in Table 4. The researchers are entrusted with the decision to assess the cost/benefit of observing a larger number of matches because an aprioristic study was agreed. So, it was decided to carry out eight observations, which is the point where the G indices exceed 0.90.

**Table 4:** Evolution of the generalization coefficient as a function of the number of matches to be observed.

**Matches**	**2**	**4**	**6**	**8**
**Categories**	**83**	**83**	**83**	**83**
**Relative G Coefficient**	**0.738**	**0.849**	**0.894**	**0.918**
**Absolute G Coefficient**	**0.737**	**0.849**	**0.894**	**0.918**

A new software called^1^ “Function Estimation” has been used in this work. This program is a software tool running on Windows operating system and developed under the .NET platform whose main objective is the estimation of the function that best fits a point cloud using different regression and interpolation techniques, as well as plotting them and calculating the absolute and relative errors made.

**Figure 1:**
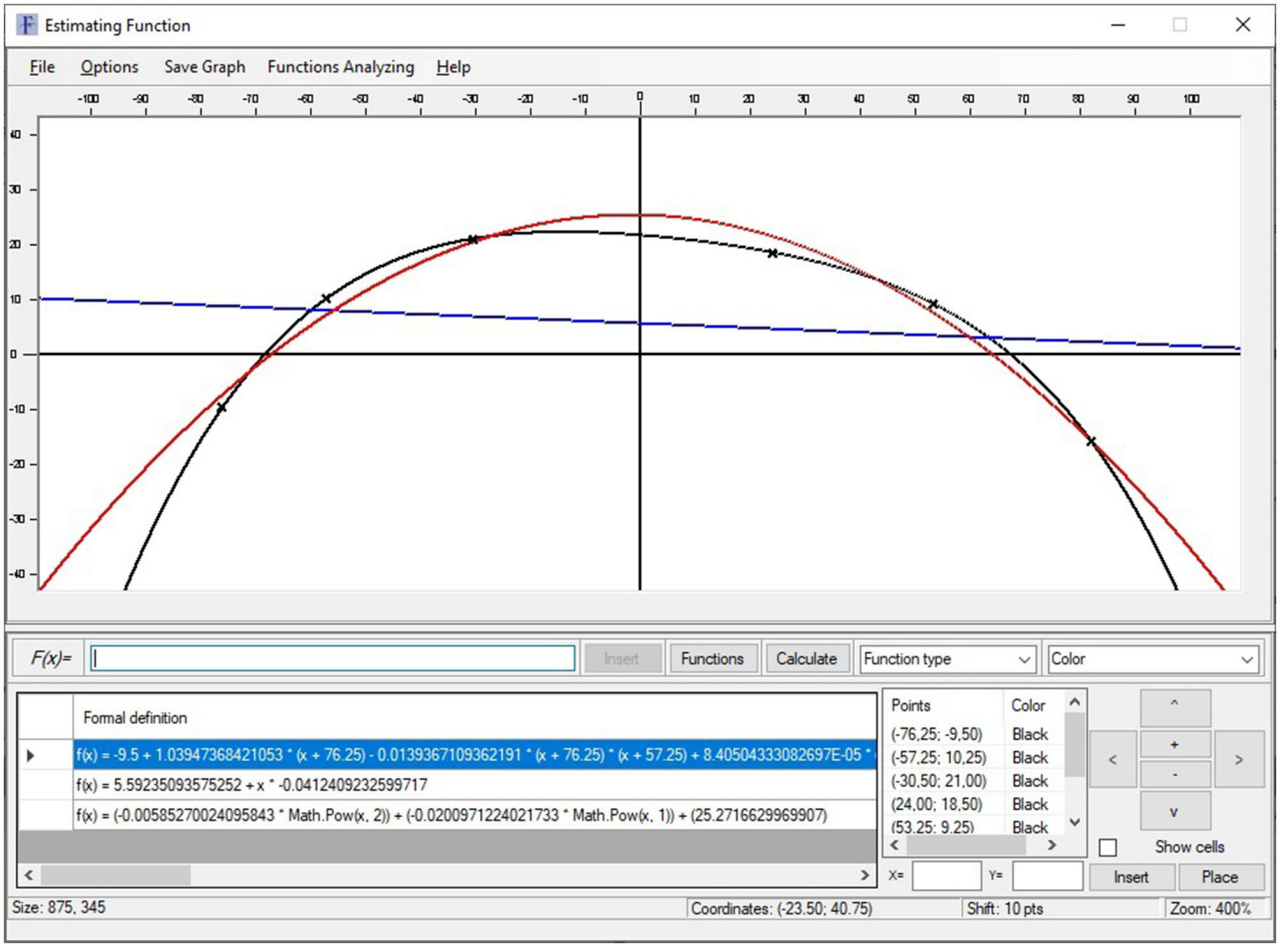
Main application screen.

This application implements linear and nonlinear regression models in the analysis of functions applied in psychology, especially applied to sports. Its versatility allows using it in any other field of knowledge where the analysis and comparison of two-dimensional functions are necessary, which makes this program a very interesting tool for any problem where you want to model and simulate the behavior of a variable where a set of points are known (Figure 1).

This software [called (see Footnote 1) “Function Estimation”] allows estimating the function that models a cloud of points. It also has methods that allow the user to choose between generating linear and nonlinear regression models. To achieve greater accuracy, it provides the possibility of generating polynomial interpolation functions, with a minimum margin of error and exact to the specified cloud of points by the Newton's divided differences method. The implemented approximation methods offered by the tool are:

- *Linear regression model:*
  ◦ *Method of least squares*

- *Nonlinear regression models*:
  ◦ *Logarithmic regression*
  ◦ *Exponential regression*
  ◦ *Polynomial interpolation (divided difference method)*

In addition to the different cloud of points approximation function calculations and plotting, the application allows saving and loading both the cloud of points and the generated functions, in a file on the local machine as well as on the MenPas platform account (www.menpas.com) (González-Ruiz et al., 2010, 2015, 2018; Pastrana et al., 2019; Reigal et al., 2020) associated to logged in user.

**Figure 2:**
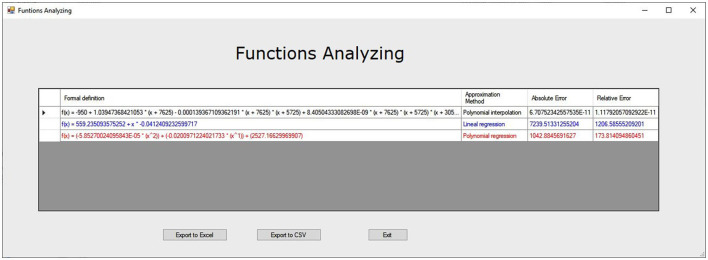
Function analysis.

It also allows the export of graph as a Joint Photographic Experts Group (JPEG) format image as well as the analysis of the functions generated, obtaining the absolute and relative errors made by each function with respect to the cloud of points (Figure 2).

### Procedures

Once the tool has been validated and the reliability of the observers has been estimated by means of the data quality and generalizability analyses, the observations of the eight matches are coded and the polar coordinate analyses have been carried out using the Hoisan program (Hernández-Mendo et al., 2012a).

First, a sequential analysis of all the observations has been made with the selected focal behavior being performed, obtaining the *Z* results with a delay range between −5 and 5. Taking those values, calculations were made to determine the Zsum parameters (prospective and retrospective), the quadrant assignment, the module, the angle, and the transformed angle (TA) for the rest of the categories (Castellano and Hernández-Mendo, 2003). Each quadrant characterization is as follows (Castellano and Hernández-Mendo, 2003):

Quadrant I [+,+]: Criterion behavior is excited with respect to mating behavior in the retrospective and prospective perspective.
Quadrant II [–,+]: The criterion behavior has a relationship with respect to the mating behavior of excitation in retrospective perspective and inhibition in prospective perspective.
Quadrant III [–,–]: The criterion behavior has a relationship with respect to inhibition mating in the retrospective and prospective perspective.
Quadrant IV [+,–]: The criterion behavior has a relationship with the mating behavior of excitation in prospective perspective and inhibition in retrospective perspective.

The behavior selected as focal (*given*) has been the one-on-one outside the zone play initiation (UxE).

Finally, an estimation of functions was carried out. The objective of regression analysis is to determine a simple mathematical function describing the behavior of a variable given the values of one or more other variables. In simple regression analysis, the aim is to study and explain the behavior of a variable, called as dependent variable or variable of interest, noted as y, from another variable, called as explanatory variable, predictor variable or independent variable, noted as *x*. This function should validate the observed values and work as a predictive model for unobserved values. The function estimation software developed allows the use of the following approximation techniques or methods:

- Linear regression. The generated function to model the observations is a line. Using the least squares method, the coefficients a and b of the equation of the line are determined: *y* = *a* + *bx*, which best fits the *n* pairs (*x*_*i*_, *y*_*i*_) observed.
- Polynomial regression. It is based on linear regression but manages to add curvature to the model by introducing new predictors obtained by raising all or some of the original predictors to different powers.
- Exponential regression. In certain occasions, the dependence between the variables *X* and *Y* is exponential, in which case it is of interest to fit to the cloud of points a function of type *y* = *e*^*bx*^ and by means of a linear transformation, taking neperian logarithms, the problem is converted into a question of linear regression: *Ln*(*y*) = *bx* +*LN*(*a*).
- Logarithmic regression. The function that is generated is a logarithmic curve = *Ln*(*x*)+*b*, which can be analyzed as a straight line, but instead of being referenced to the original variables *X* and *Y*, it is referenced to *Ln*(*x*) and *Y*.
- Polynomial interpolation. This is a nonlinear regression that uses a polynomial of degree n to estimate a function of the type $y=\;\overset{n}{\underset{i=0}{\sum }}a_{i}x^{i}$. To solve this function, the tool uses Newton's divided differences method (Beltrán Álvarez, 2019). This approximation method manages to obtain a polynomial representing the approximation of the points displayed in the panel. This function has a minimum margin of error as this is a characteristic of this interpolation method.

## Statistical Analysis

### Polar Coordinates

The polar coordinates analysis shows the results obtained from the UxE focal category: one-on-one outside the zone play initiation. Table 5 shows the relationship between this focal behavior and the rest of the mating behaviors for all the observations made.

**Table 5:** Significant relationships and vector representation between outdoor 1x1 focal behavior (UxE) and mating behaviors for all observations.

**Category**	**Quadrant**	**P.Prospective**	**P.Retrospective**	**Ratio**	**Radio**	**Angle**
Location_V	I	4.55	3.67	0.63	5.84	38.85
Marker_PU7	I	2.23	3.22	0.82	3.92	55.31
Action_Zone_FZI	I	1.85	0.93	0.45	2.07	26.72
Cont_JLN	I	0.37	2.62	0.99	2.65	82.07
Action_Zone_TLD	II	−1.92	1.43	0.6	2.39	143.25
Location_L	III	−4.55	−3.67	−0.63	5.84	218.85
Marker_GM7	III	−2.06	−0.15	−0.07	2.07	184.24
Action Zone_DZI	III	−0.51	−1.95	−0.97	2.01	255.37
Action Zone_DZD	III	−0.11	−2.04	−1	2.05	267
Cont_TO	IV	0.61	−1.95	−0.95	2.04	287.3
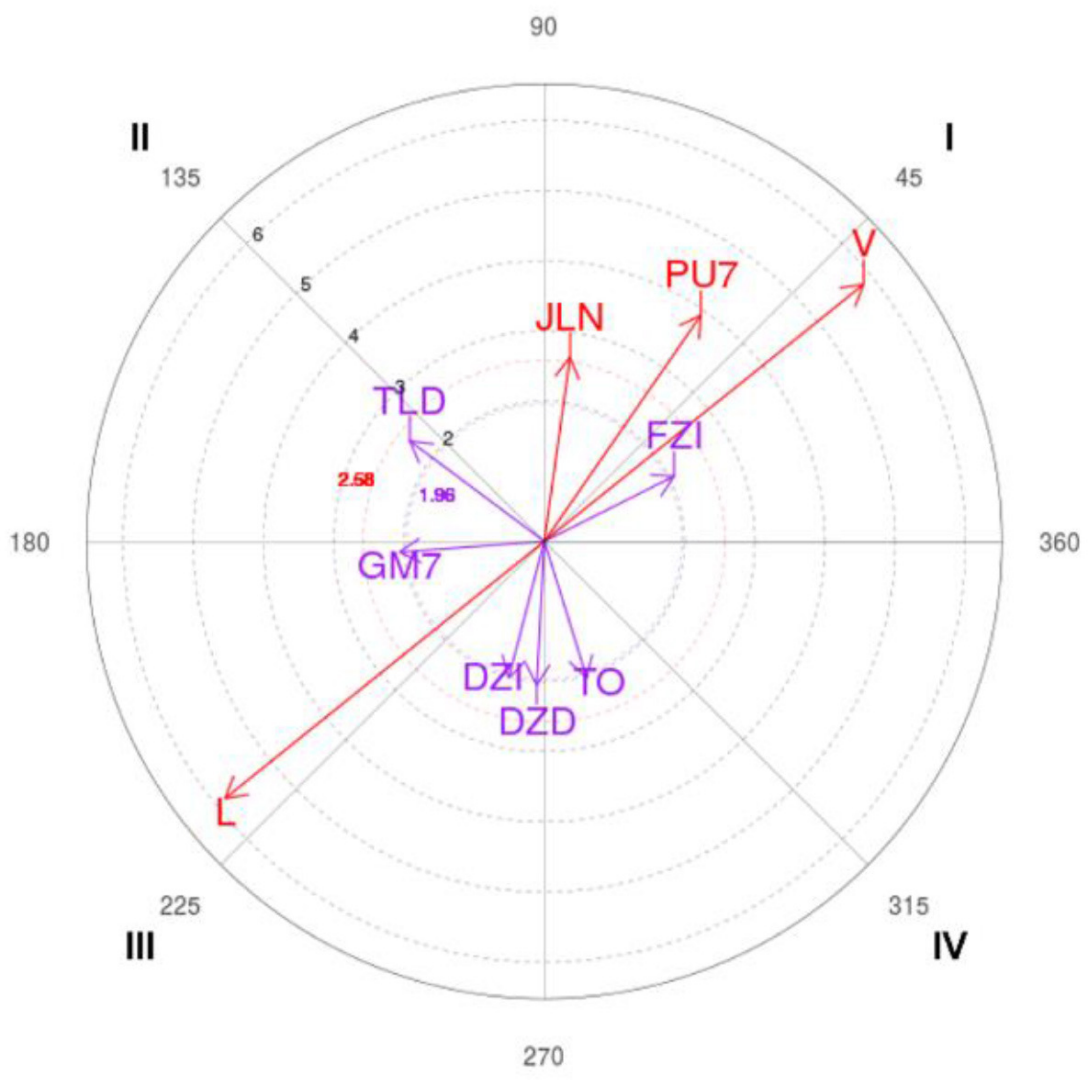						

### Function Estimation

#### Quadrant I: Focus Behavior UxE

It can be seen that polynomial interpolation is the only acceptable model with an absolute error order of 10^−13^ and a relative error for each observation of the order of 10^−14^. The rest of the models present an absolute error and a relative error for each observation of the order of unity and are therefore not adequate (Table 6).

**Table 6:** Function estimation quadrant I: focus behavior UxE.

**Formal definition**	**Approximation method**	**Absolute error**	**Relative error**
f(*x*) = 1.83894777847031 + *x* * 0.34268987623542	Linear regression	3.085848099	0.771462025
f(*x*) = [0.20606049394221615 * (x^2^)] + [−0.709708643139346 * (x^1^)] + (2.70089123951601)	Polynomial regression	2.461177597	0.615294399
f(*x*) = 1.72153168717492 * (1.14102197932492^x^)	Exponential regression	3.521748478	0.880437119
f(*x*) = 0.312405123672677 * Log(*x*) + (2.45863486091638)	Logarithmic regression	3.441644013	0.860411003
f(*x*) = 2.62−1.14189189189189189 * (*x*−0.37) + 3.85387509750838 * (*x*−0.37) * (*x*−1.85) − 1.43875713587674 * (*x* − 0.37) * (*x* − 1.85) * (*x* − 2.23)	Polynomial interpolation	1.59983E−13	3.99958E−14
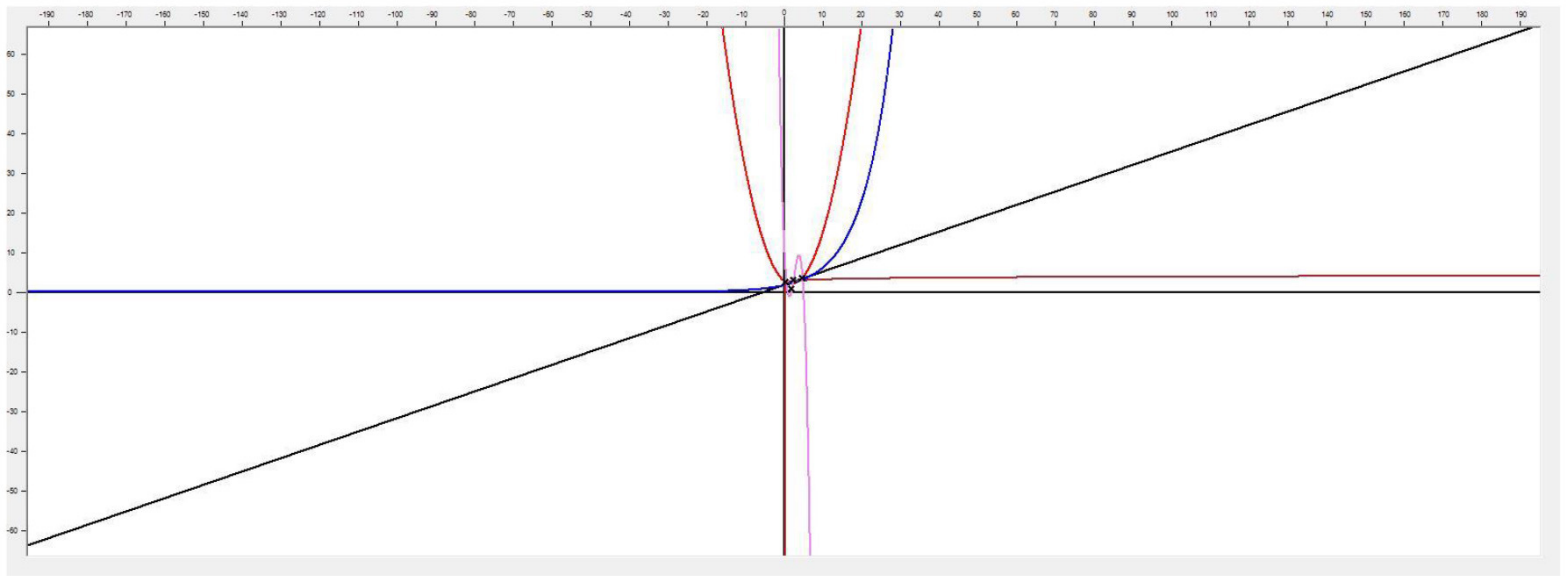			

#### Quadrant II: UxE Focal Conduct

As quadrant II has only one significant vector, the only possible model is a linear regression passing through the origin (Table 7).

**Table 7:** Function estimation quadrant II: UxE focal conduct.

**Formal definition**	**Approximation method**	**Absolute error**	**Relative error**
f(*x*) = 0 + *x* * −0.74402020404308778	Linear regression	2.22E−16	1.11E−16
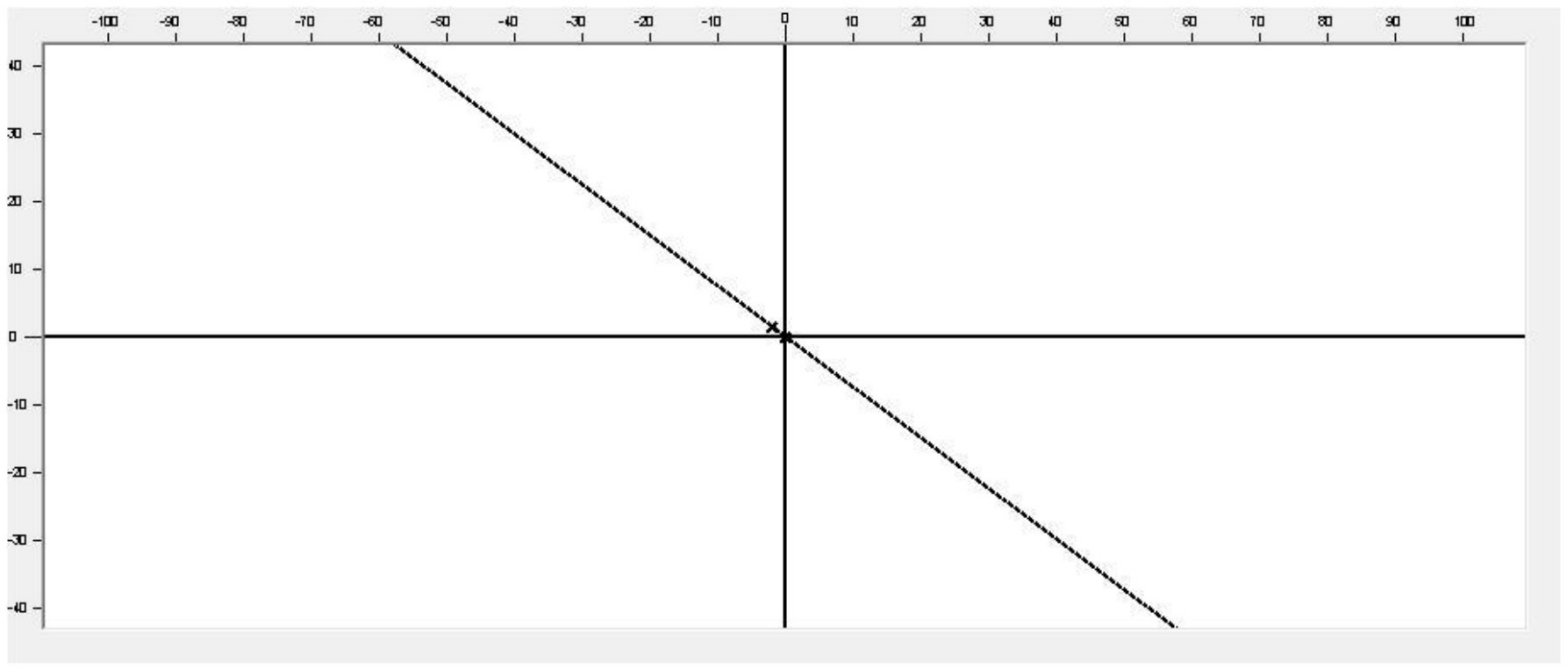			

This model has a practically 0 error for the observation made but it is not very suitable for predictive purposes because it is based on a single significant vector.

#### Quadrant III: Focus Behavior UxE

It can be seen how polynomial interpolation offers the only acceptable model with an absolute error of the order of 10^−14^ and a relative error for each observation of the order of 10^−14^. The linear and polynomial regression models present an absolute error and a relative error for each observation of the order of unity and are therefore not adequate (Table 8).

**Table 8:** Function estimation quadrant III: focus behavior UxE.

**Formal definition**	**Approximation method**	**Absolute error**	**Relative error**
f(*x*) = −1.3411038820748 + *x* * 0.338255113651562	Linear regression	3.775818832	0.943954708
f(*x*) = [−0.540506071985558 * (*x*^2^)] + [−2.21923904436031 * (*x*^1^)] + (−2.55611350389291)	Polynomial regression	0.813419944	0.203354986
Calculation is not feasible if there is a point <0	Exponential regression		
Calculation is not feasible if there is a point <0	Logarithmic regression		
f(*x*) = −3.67 + 1.4136546184739 * (*x* + 4.55) – 0.637362609171916 * (*x* + 4.55) * (*x* + 2.06) + 0.251691777600587 * (*x* + 4.55) * (*x* + 2.06) * (*x* + 0.51)	Polynomial interpolation	4.82669E−14	1.20667E−14
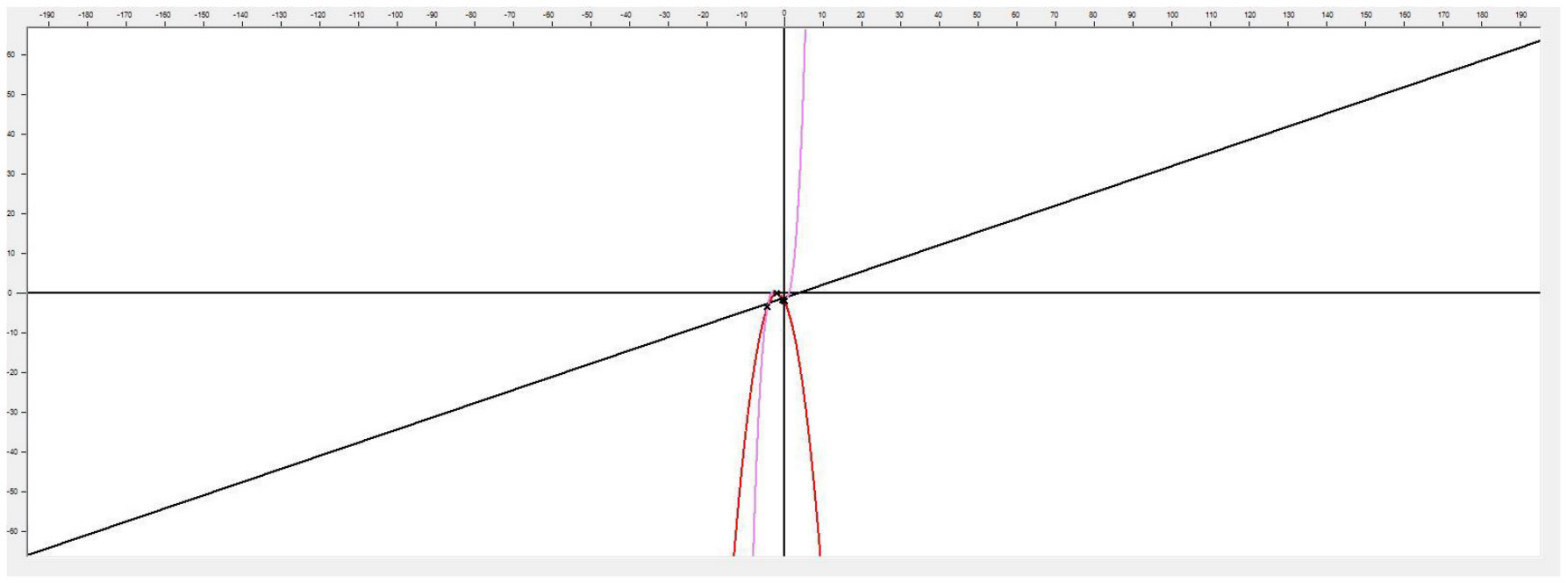			

#### Quadrant IV: Focus Behavior UxE

It can be seen that polynomial interpolation is the only acceptable model with an absolute error of the order of 10^−15^ and a relative error for each observation of the order of 10^−16^. The rest of the models present an absolute error and a relative error for each observation in the unity order, so they are not adequate (Table 9).

**Table 9:** Function estimation quadrant IV: focus behavior UxE.

**Formal definition**	**Approximation method**	**Absolute error**	**Relative error**
f(*x*) = −2.2063070709768096 + *x* * 0.937104708362614	Linear regression	0.074546732	0.024848911
f(*x*) = [0.200742240215876 * (*x*^2^)] + [0.490215924426558 * (*x*^1^)] + (−2.15635273279353)	Polynomial regression	2.06501E−14	6.88338E−15
Calculation is not feasible if there is a point <0	Exponential regression		
f(*x*) = 0.63224848268029558 * Log(*x*) + (−0.707156373780864)	Logarithmic regression	0.18847619	0.062825397
f(*x*) = −2.1 + 0.915789473684211 * (*x* – 0.11) + 0.200742240215924 * (*x* – 0.11) * (*x* – 2.01)	Polynomial interpolation	1.47105E−15	4.90349E−16
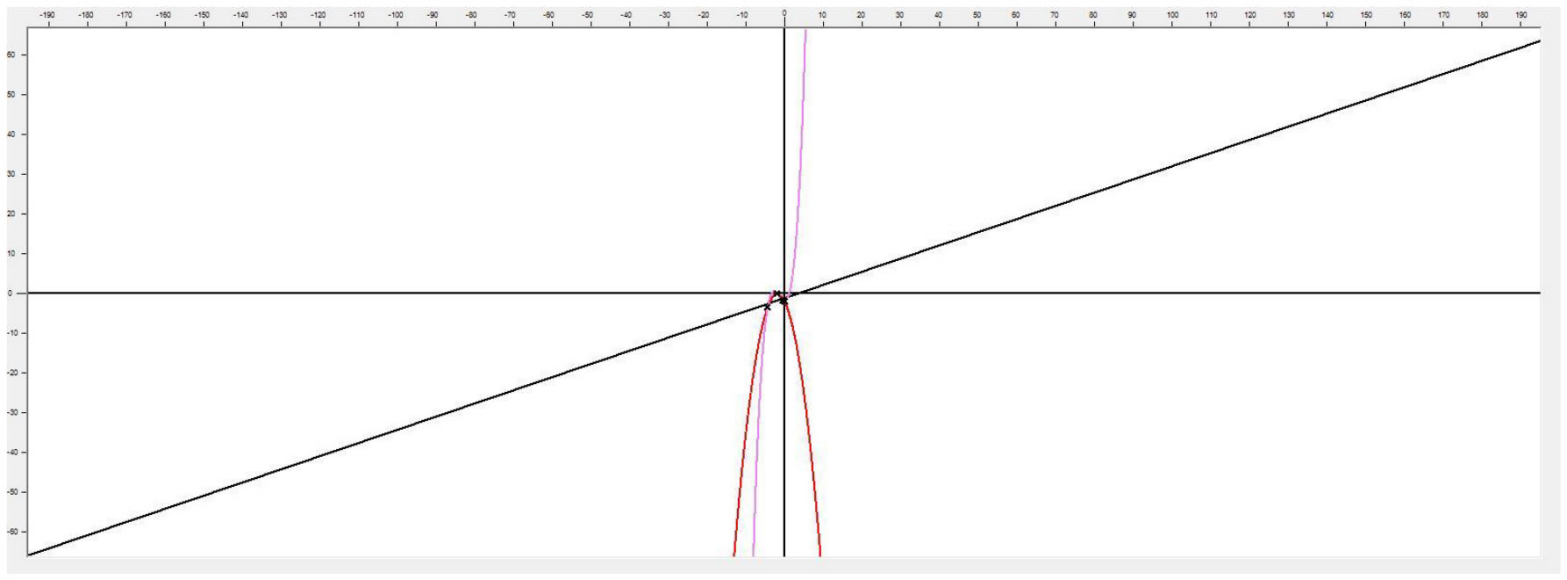			

## Results

### Polar Coordinates

Polar coordinates analysis results for the one-on-one outside the zone play initiation focal behavior (UxE) show that conditioned behaviors V (Visitor), PU7 (Losing by 7 or less), FZI (Outside Left Zone), and JLN (continuation Free Play No advantage) in quadrant I, where the established relationship is a mutual excitation in both perspectives. In the second quadrant, with a relationship of inhibition in the prospective perspective and excitation in the retrospective one, only the action zone behavior TLD (Triple Right Side) manifests itself as significant; while in quadrant III, several mating behaviors appear again: L (local), GM7 (gaining of more than 7), DZI (within zone left), and DZD (within zone right); behaviors that are related to the focal category with mutual inhibition in both perspectives. Finally, in quadrant IV, where a relationship of excitation in the prospective perspective and inhibition in the retrospective perspective is established, only the TO (shooting with opposition) behavior shows a significant relationship.

### Function Estimation

There are two options for the function estimation modeling the behavior of the variables calculation: on one hand, all the generated vectors in the polar coordinates analysis can be taken or on the other hand, taking only those that are significant. Using only the significant ones would generate a simpler function, but could increase the error generated in the interpolation. To determine whether this increase in error is significant and so undesirable, the percentage of error made with respect to the interval of possible values has been calculated, that means, determining how significant the increase in error committed is, because an absolute 0.1 error is not the same for values in the range from 0 to 100 than values in the range from 0 to 1.

This calculation has allowed us to determine that when there are many significant vectors (case quadrant I), we have many points corresponding to the vectors, so, the error increase in the nonsignificant points when using only the significant vectors approximation is less than 1% (0.48% on an average). However, in quadrant II, since there was only one significant vector, the position (0,0) had to be assumed, i.e., no stimulus implies no response. In this case, when using only the significant vectors approximation error increase is 0.32% for significant vectors but error increases on an average by 38% for the nonsignificant. Therefore, it can be concluded that when there are few significant vectors, all of them should be used to calculate the function estimation, but when there are many significant vectors, it would be enough using only the significant vectors.

## Discussion

This study, carried out by observing games of a professional basketball team in the Spanish league (ACB), has the objective of analyzing the relationships between the behaviors occurring when using individual technical-tactical means at the beginning of the transition phase and the defense carrying out the defensive balance.

Data quality and generalizability analyses results show that the design of the *ad hoc* tool is correct and it allows the reliable recording of the actions under study. Polar coordinates analysis has been shown to be a useful technique for the exploration of the relationships established between the behaviors of a tactical situation in competitive sport as it has also been shown in other collective sports (Vázquez-Diz et al., 2019a,b). Thus, the results of polar coordinate analysis have shown statistically significant mating behaviors in the onset with outside one-on-one play.

Although transitions take about only 10% of all game possessions (Cruz and Tavares, 1998; Fernandes and Tavares, 2004), according to Cruz and Tavares (1998), winning teams have a significantly higher percentage of transitions in the game compared with losing teams. In addition, Gómez (2007) observed an efficiency of around 60% in transitions. Therefore, its study can be very interesting to optimize the play and achieve a victory.

The results found seem to indicate that the observed team, Unicaja de Málaga, does not play enough this type of game by concepts and is less predictable, so it could become a more predictable team from the tactical point of view and, so, easier to analyze by the rivals. From this point of view, this *mixed method* study allows us to explore the tactical behaviors of the teams defining intervention procedures that will optimize resources during training for the competition (Jiménez-Salas et al., 2020b), showing indications of behaviors that allow to make tactical interpretation of the game and the definition of intervention programs to improve the performance in the team (Vázquez-Diz et al., 2019a).

Direct blocking as a tactical means to create and benefit from a greater advantage produced by a defensive disorganization is one of the most used tactical means in the ACB League and it is the second most used means for finishing an action in transitions (Romarís-Durán, 2016). The one-on-one at the beginning of this phase of the game has been shown in more than 15% of the transitions in the ACB League; and just like the direct block, this action is used to obtain advantage finishing the play, being more used in the outside the zone play than in the inside the zone play, because transitions without inside the zone play obtain better results than transitions with inside the zone play (Romarís Durán et al., 2013; Serna-Bardavío et al., 2017).

The results obtained after the polar coordinates analysis with the focal behavior one-on-one outside the zone show a clearly defined tendency. Relationships of mutual excitement are established with a tight score but not managing to overcome the opponent, so those actions lead to situations without tactical advantage that support an offensive success achievement, executing outside the left zone, which is consistent with the results obtained in other studies where the most used areas were outside of the zone and the sides (Muñoz-Arroyave et al., 2015; Serna-Bardavío et al., 2017). On the other hand, the one-on-one outside of the zone presents a mutual inhibition relationship with the play inside the zone as well as an unbalanced score in favor.

The findings of this research can be used by coaches and trainers, from both, teams and national teams, in order to analyze the possible patterns in the play of the opponents they are going to face, with the aim of improving the level of preparation of their own players against those opponents. Those data could help in the design and planning of training sessions where those patterns are simulated as well as the best way to counteract them. For all these reasons, it would be of great interest to continue exploring this researching line, in order to have more and more studies to compare the results with. Function estimation combined with polar coordinate analysis is a powerful and novel tool allowing to estimate from the focal category (UxE in this case), the relationship between this focal behavior and the rest of the mating behaviors for possible future observations.

This study, focused on only one team, has shown all the virtues in the analysis technique; however, it also shows limitations that should be considered, such as the impossibility of contrasting the information obtained with highly relevant facets of the game, as it could be the receiver of a possible rebound or the reception place for the first pass. Limitations that could be solved by designing new observation tools focused on the other phases in the play, proposals that become future lines of research for this sport, as well as applying them to several national and/or international competitions in different categories and/or gender to explore how they behave in those areas.

## Data Availability Statement

The raw data supporting the conclusions of this article will be made available by the authors, without undue reservation.

## Author Contributions

AH-M, VM-S, RR-G, JP-B, RL, BT-G, and JV-D participated in the study design and data collection, performed statistical analyses and contributed to the interpretation of the results, wrote the manuscript, approved the final manuscript as submitted, and reviewed and provided feedback to the manuscript. All authors made substantial contributions to the final manuscript.

## Conflict of Interest

The authors declare that the research was conducted in the absence of any commercial or financial relationships that could be construed as a potential conflict of interest.

## Publisher's Note

All claims expressed in this article are solely those of the authors and do not necessarily represent those of their affiliated organizations, or those of the publisher, the editors and the reviewers. Any product that may be evaluated in this article, or claim that may be made by its manufacturer, is not guaranteed or endorsed by the publisher.

## Footnotes

^1^This program can be downloaded from the MenPas Psychosocial Assessment Platform (www.menpas.com) from the Work Area II in Decision Making (http://150.214.108.138/menpas/Eval_Individual/Descarga%20E%20Funciones.aspx). All programs are free of charge, platform registration is just needed.
